# Impact of Proteinuria on Renal Outcomes in the BALANCE Trial

**DOI:** 10.1016/j.ekir.2025.11.016

**Published:** 2025-11-19

**Authors:** Eric L. Wallace, Christoph Wanner, Giovanni Piotti, David Lee, Irene Koulinska, Raul Chertkoff, David G. Warnock, BALANCE Study Group, Eric L. Wallace, Ozlem Goker-Alpan, William R. Wilcox, Myrl Holida, John Bernat, Nicola Longo, Aleš Linhart, Derralynn A. Hughes, Robert J. Hopkin, Camilla Tøndel, Mirjam Langeveld, Pilar Giraldo, Antonio Pisani, Dominique Paul Germain, Ankit Mehta, Patrick B. Deegan, Maria Judit Molnar, Damara Ortiz, Ana Jovanovic, Michael Muriello, Bruce A. Barshop, Virginia Kimonis, Bojan Vujkovac, Albina Nowak, Tarekegn Geberhiwot, Ilkka Kantola, Jasmine Knoll, Stephen Waldek, Khan Nedd, Amel Karaa, David G. Warnock

**Affiliations:** 8 Department of Medicine, Division of Nephrology, The University of Alabama at Birmingham, Birmingham, Alabama, USA; 9 Lysosomal and Rare Disorders Research and Treatment Center, Inc, Fairfax, Virginia, USA; 10 Department of Human Genetics, Emory University School of Medicine, Atlanta, Georgia, USA; 11 Department of Pediatrics, University of Iowa Hospitals and Clinics, Iowa City, Iowa, USA; 12 Department of Pediatrics, University of Iowa Hospitals and Clinics, Iowa City, Iowa, USA; 13 Department of Pediatrics, Division of Medical Genetics, University of Utah Health, Salt Lake City, Utah, USA; 14 Department of Internal Medicine, School of Medicine, Charles University and General University Hospital, Prague, Czech Republic; 15 Lysosomal Storage Disorders Unit, Royal Free London NHS Foundation Trust and University College London, London, UK; 16 Department of Human Genetics, Cincinnati Children's Hospital Medical Center, Cincinnati, Ohio, USA; 17 Department of Clinical Science, University of Bergen, Bergen, Norway and Department of Pediatrics, Haukeland University Hospital, Bergen, Norway; 18 Academisch Medisch Centrum Universiteit van Amsterdam, Amsterdam, The Netherlands; 19 Unidad de Investigación Traslacional. Hospital Universitario Miguel Servet, Instituto de Investigación Sanitaria Aragón, Zaragoza, Spain and Centro de Investigación Biomédica en Red de Enfermedades Raras, Zaragoza, Spain; 20 Department of Public Health, Universita degli Studi di Napoli Federico II, Napoli, Italy; 21 Division of Medical Genetics, University of Versailles, Garches, France; 22 Baylor University Medical Center at Dallas, Dallas, Texas, USA; 23 Lysosmal Disorders Unit, Department of Medicine, Cambridge University Hospitals NHS Foundation Trust, Cambridge, UK; 24 Institute of Genomic Medicine and Rare Disorders, Semmelweis University Clinical Center, Budapest, Hungary; 25 Department of Pediatrics, UPMC Children’s Hospital of Pittsburgh, Pittsburgh, Pennsylvania, USA; 26 Mark Holland Metabolic Unit, Northern Care Alliance NHS Foundation Trust, Greater Manchester, UK; 27 Department of Pediatrics, Medical College of Wisconsin, Milwaukee, Wisconsin, USA; 28 Department of Pediatrics, University of California San Diego, La Jolla, California, USA; 29 Department of Pediatrics, University of California Irvine, Irvine, California, USA; 30 Department of Internal Medicine, General Hospital Slovenj Gradec, Slovenj Gradec, Slovenia; 31 Department of Endocrinology and Clinical Nutrition, University Hospital Zurich and University of Zurich, Zurich, Switzerland; 32 Department of Diabetes, Endocrinology and Metabolism, Queen Elizabeth Hospital Birmingham, Birmingham, UK; 33 Division of Medicine, Turku University Hospital, Turku, Finland; 34 Phoenix Children’s Hospital, Phoenix, Arizona, USA; 35 University of Sunderland, Sunderland, UK; 36 Infusion Associates, Grand Rapids, Michigan, USA; 37 Massachusetts General Hospital for Children, Boston, Massachusetts, USA; 38 Department of Medicine, Division of Nephrology, The University of Alabama at Birmingham, Birmingham, Alabama, USA; 1 Division of Nephrology, University of Alabama at Birmingham, Birmingham, Alabama, USA; 2 Department of Clinical Research and Epidemiology, Comprehensive Heart Failure Center, University Hospital Würzburg, Würzburg, Germany; 3 Chiesi Farmaceutici S.p.A., Parma, Italy; 4 Chiesi Canada Corp., Woodbridge, Ontario, Canada; 5 Chiesi USA, Inc., Boston, Massachusetts, USA; 6 Protalix Biotherapeutics, Carmiel, Israel

## Introduction

Pegunigalsidase alfa is a PEGylated α-galactosidase A enzyme replacement therapy approved for the treatment of Fabry disease.^1^ In the phase 3 head-to-head BALANCE trial (NCT02795676), patients with Fabry disease who had received agalsidase beta for ≥ 1 year and had declining kidney function at screening (annualized estimated glomerular filtration rate [eGFR] slope ≤ −2.0 ml/min per 1.73 m^2^/yr) were randomly assigned 2:1 to receive pegunigalsidase alfa or continue agalsidase beta for 2 years. Randomization was stratified based on urine protein-to-creatinine ratio (UPCR) (< 1 or ≥ 1 g/g). In the prespecified analysis, the primary end point was to assess noninferiority in renal efficacy between treatment arms, measured by median annualized eGFR slope differences (pegunigalsidase alfa − agalsidase beta). Annualized eGFR slopes were calculated using the Chronic Kidney Disease Epidemiology Collaboration (CKD-EPI) serum creatinine (2009) equation and analyzed using quantile regression with treatment arm as a covariate. The primary end point was met; at 2 years, pegunigalsidase alfa demonstrated noninferiority to agalsidase beta with an eGFR slope difference between treatment arms of −0.4 ml/min per 1.73 m^2^/yr (95% confidence interval [CI] −2.4 to 1.7) and the lower limit of the treatment difference CI was above the predefined noninferiority margin (−3.0 ml/min per 1.73 m^2^/yr).^2^

In the primary analysis, although the proportions of patients with baseline UPCR ≥ 1 g/g were balanced between the pegunigalsidase alfa and agalsidase beta arms (13.7% vs. 12.0%, respectively), a greater proportion of patients randomized to pegunigalsidase alfa (31% vs. 20%, respectively) had a baseline UPCR > 0.5 g/g, and median baseline UPCR was > 50% higher with pegunigalsidase alfa (0.13 vs. 0.07 g/g, respectively).^2^^,^^3^ Given that proteinuria is a strong predictor of eGFR decline,^4^^,^^5^ a *post hoc* analysis was conducted to explore the impact of baseline UPCR on eGFR outcomes. Specifically, baseline UPCR was modeled as a continuous variable, rather than the binary threshold used in the primary analysis, to assess its effect on annualized eGFR slope.^6^ In addition, the concurrent collection and measurement at the central laboratory of serum creatinine and serum cystatin C allowed a comprehensive *post hoc* assessment of eGFR slope using the CKD-EPI equation based on the combination of creatinine and cystatin C (2012),^7^ which has been shown to provide greater accuracy than equations based on creatinine or cystatin C alone.^8^

## Results

In this *post hoc* analysis, the eGFR slope for each patient was calculated using linear regression. Mean eGFR slope was estimated using analysis of covariance and median eGFR slope by quantile regression. Baseline UPCR was included as a continuous covariate in the quantile regression model. The treatment effect on annualized eGFR slopes was expressed as the difference between the treatment arms.

Of the 77 patients treated in BALANCE, 76 were included in this analysis (pegunigalsidase alfa, *n* = 51 [2 patients had cystatin C data at all assessment points except baseline]; agalsidase beta, *n* = 25). Baseline characteristics are shown in Supplementary Table S1. Baseline eGFR values (ml/min per 1.73 m^2^) were similar between treatment arms (mean: 73.9 vs. 74.2; median: 73.9 vs. 74.9 for pegunigalsidase alfa vs. agalsidase beta) with comparably broad ranges (30.2–125.9 vs. 34.1–107.6, respectively). Mean baseline eGFR slopes were balanced between treatment arms (−8.0 vs. −8.2 ml/min per 1.73 m^2^/yr), although baseline variation was greater in the pegunigalsidase alfa than the agalsidase beta arm (SD: 6.7 and 4.3, respectively).

**Figure 1:**
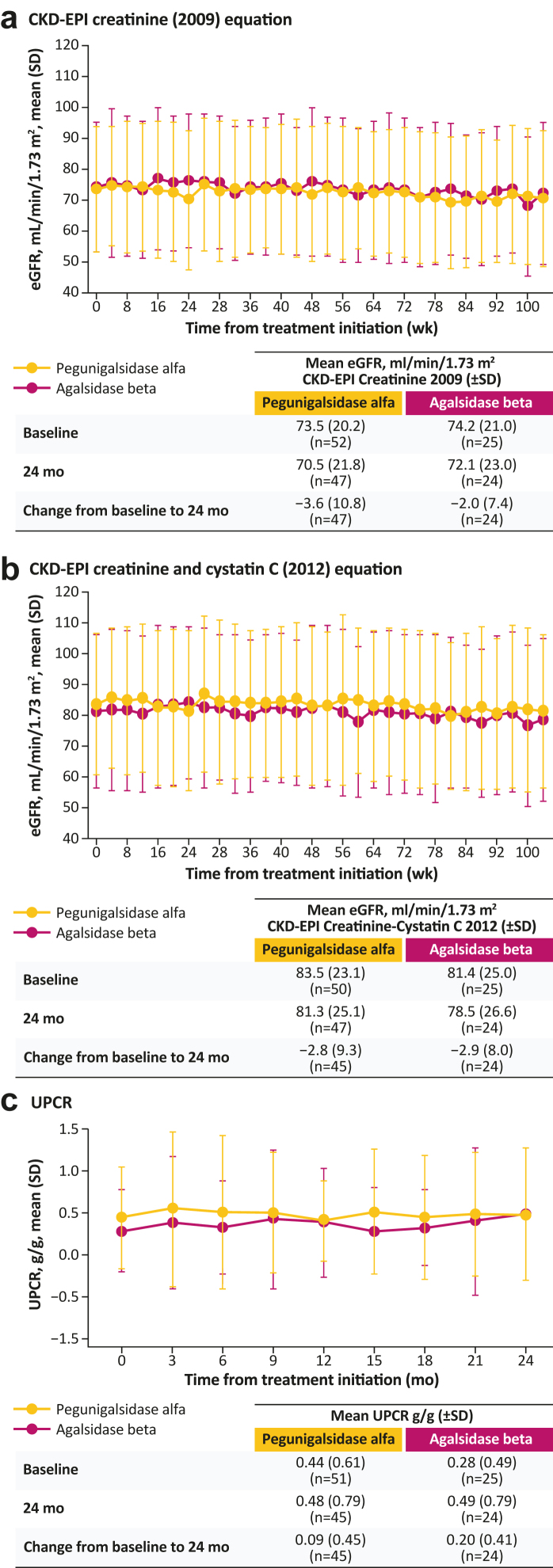
Change over time in (a) eGFR calculated using the CKD-EPI creatinine (2009) equation, (b) eGFR calculated using the CKD-EPI creatinine and cystatin C (2012) equation, and (c) UPCR, in patients treated with pegunigalsidase alfa or agalsidase beta. Changes from baseline to 24 months are based on the patients who have had data collected at both time points. CKD-EPI, Chronic Kidney Disease Epidemiology Collaboration; eGFR, estimated glomerular filtration rate; UPCR, urine protein-to-creatine ratio.

Over the 24-month treatment period, changes in eGFR were similar between treatment arms irrespective of the equation used to calculate eGFR (Figure 1a and b). UPCR remained relatively stable over time in each arm (Figure 1c), with mean (SD) changes from baseline to 24 months of 0.09 (0.45) g/g for pegunigalsidase alfa and 0.20 (0.41) g/g for agalsidase beta. Renal function stabilized in both arms, despite declining renal function at baseline.

**Figure 2:**
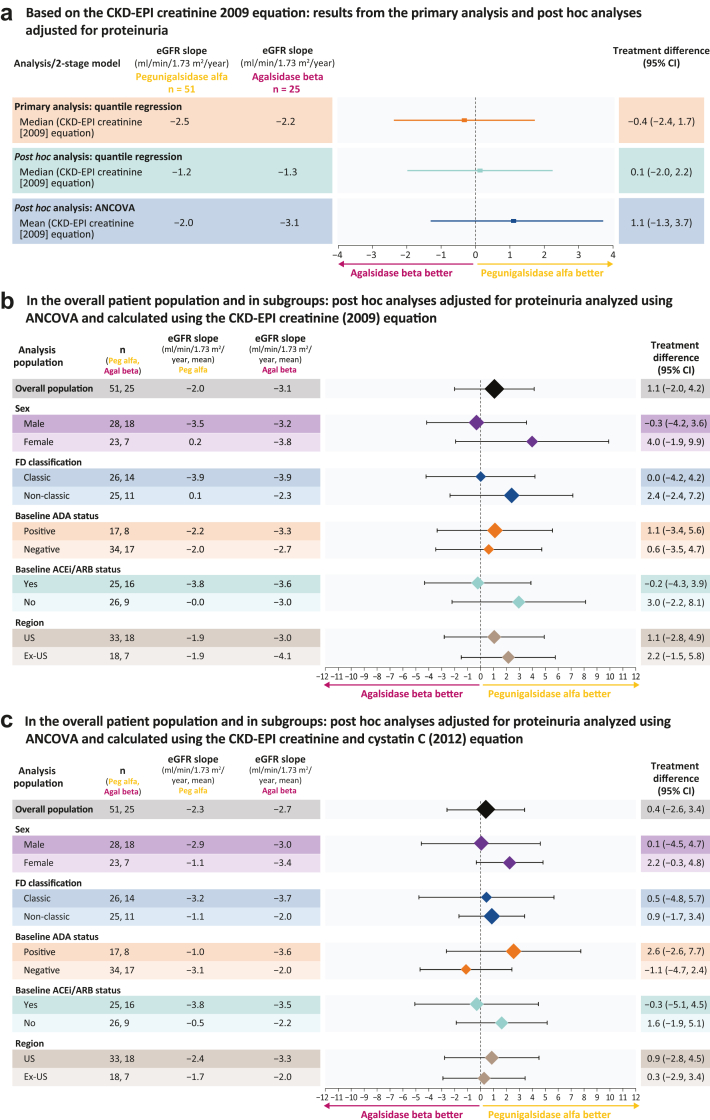
Treatment differences in annualized eGFR slope. (a) Based on the CKD-EPI creatinine (2009) equation: results from the primary analysis and *post hoc* analyses adjusted for proteinuria. (b) In the overall patient population and in subgroups: *post hoc* analyses adjusted for proteinuria analyzed using ANCOVA and calculated using the CKD-EPI creatinine (2009) equation. (c) In the overall patient population and in subgroups: *post hoc* analyses adjusted for proteinuria analyzed using ANCOVA and calculated using the CKD-EPI creatinine and cystatin C (2012) equation. ACEi, angiotensin-converting enzyme inhibitor; ADA, antidrug antibody; Agal, agalsidase; ANCOVA, analysis of covariance; ARB, angiotensin II receptor blocker; CI, confidence interval; CKD-EPI, Chronic Kidney Disease Epidemiology Collaboration; eGFR, estimated glomerular filtration rate; FD, Fabry disease; peg, pegunigalsidase.

With adjustment for baseline UPCR as a continuous variable, no statistically significant differences in annualized eGFR slopes were observed between treatment arms, whether assessed by quantile regression or analysis of covariance (Figure 2a). These findings are consistent with the BALANCE primary analysis (as well as sex-adjusted *post hoc* analysis), reaffirming comparable treatment effects in annualized eGFR slopes. Point estimates showed a positive treatment difference with pegunigalsidase alfa (+0.1 with quantile regression and +1.1 with analysis of covariance), but neither reached statistical significance (Figure 2). Similar outcomes were observed when eGFR slopes were calculated using the CKD-EPI equation based on creatinine (2009) alone, or the combination of creatinine and cystatin C (2012) (Supplementary Figure S1). Across all models, the 95% CI for the treatment difference included 0, reinforcing the conclusion of comparable efficacy. Additional subgroup analyses with UPCR as a continuous variable, analyzed using analysis of covariance and calculated using CKD-EPI equations based on creatinine ± cystatin C further confirmed these findings across patient subgroups with different UPCR profiles (Figure 2b and c). In addition, it is noteworthy that though adjusting for UPCR in subgroup analyses did not result in statistically significant changes in eGFR slope differences, a numerical reduction in the slope difference was observed in some subgroups, particularly males, relative to the original analysis. In males, the eGFR slope difference between treatment arms calculated using the CKD-EPI equation based on creatinine (2009) was −0.3 ml/min per 1.73 m^2^/yr (95% CI: −3.1 to 2.8). In females, the eGFR slope difference between treatment arms calculated using the CKD-EPI equation based on creatinine (2009) was 4.0 ml/min per 1.73 m^2^/yr (95% CI: 0.3–8.7).

## Discussion

*Post hoc* analyses of annualized eGFR slope over 2 years of treatment with adjustment for baseline proteinuria as a continuous variable confirmed the primary findings from BALANCE demonstrating comparable efficacy between pegunigalsidase alfa and agalsidase beta. The additional analyses further highlighted the comparability in treatment effect on kidney function decline for pegunigalsidase alfa versus algalsidase beta, with numerical differences likely driven by the inherent variability of the outcome measure along with adjustment for UPCR as a confounder.

Controlling for proteinuria and using the eGFR equation based on both creatinine and cystatin C strengthened the reliability of these findings. When calculating eGFR, the CKD-EPI equation based on creatinine and cystatin C was recently shown to have less bias than the CKD-EPI equation based on creatinine or cystatin C alone, and the highest correct classification throughout a wide range of body mass indices.^9^

Study limitations included the BALANCE eligibility criteria; patients required a history of ≥ 1 year of agalsidase beta treatment and evidence of renal decline, which may restrict the generalizability of these findings to treatment-naïve patients or those with stable renal function. Longer-term follow-up may be necessary to assess the sustained and respective impacts of treatment on renal decline, particularly in the context of a chronic, progressive disorder. The *post hoc* nature of the analysis may be considered a limitation; however, the prospective collection of creatinine and cystatin C at the same time points with concurrent analysis at the central laboratory should have mitigated against potential bias associated with retrospective data handling and analysis.

In conclusion, the results of these *post hoc* analyses confirm the primary findings from BALANCE, and collectively provide additional evidence supporting the comparable impact on renal function in patients who switched from agalsidase beta to pegunigalsidase alfa versus those who continued on agalsidase beta, underscoring the value of comprehensive kidney function assessment in evaluating treatment outcomes in Fabry disease.

## Appendix

This manuscript presents a *post hoc* analysis of data collected as part of the BALANCE clinical trial (NCT02795676), conducted by the BALANCE Study Group. The BALANCE Study Group is a collaborative network of investigators who contributed to the original clinical trial, including its study design, patient recruitment and data collection. For the purposes of this *post hoc* analysis, a subgroup of authors within the BALANCE Study Group contributed to the conception, data analysis, interpretation of findings, and drafting of the manuscript. The authors gratefully acknowledge the contributions of the full BALANCE Study Group: Eric L Wallace (Department of Medicine, Division of Nephrology, The University of Alabama at Birmingham, Birmingham, Alabama, USA); Ozlem Goker-Alpan (Lysosomal and Rare Disorders Research and Treatment Center, Inc, Fairfax, Virginia, USA); William R Wilcox (Department of Human Genetics, Emory University School of Medicine, Atlanta, Georgia, USA); Myrl Holida (Department of Pediatrics, University of Iowa Hospitals and Clinics, Iowa City, Iowa, USA); John Bernat (Department of Pediatrics, University of Iowa Hospitals and Clinics, Iowa City, Iowa, USA); Nicola Longo (Department of Pediatrics, Division of Medical Genetics, University of Utah Health, Salt Lake City, Utah, USA); Aleš Linhart (Department of Internal Medicine, School of Medicine, Charles University and General University Hospital, Prague, Czech Republic); Derralynn A Hughes (Lysosomal Storage Disorders Unit, Royal Free London NHS Foundation Trust and University College London, London, UK); Robert J Hopkin (Department of Human Genetics, Cincinnati Children's Hospital Medical Center, Cincinnati, Ohio, USA); Camilla Tøndel (Department of Clinical Science, University of Bergen, Bergen, Norway and Department of Pediatrics, Haukeland University Hospital, Bergen, Norway); Mirjam Langeveld (Academisch Medisch Centrum Universiteit van Amsterdam, Amsterdam, The Netherlands); Pilar Giraldo (Unidad de Investigación Traslacional. Hospital Universitario Miguel Servet, Instituto de Investigación Sanitaria Aragón, Zaragoza, Spain and Centro de Investigación Biomédica en Red de Enfermedades Raras, Zaragoza, Spain) Antonio Pisani (Department of Public Health, Universita degli Studi di Napoli Federico II, Napoli, Italy); Dominique Paul Germain (Division of Medical Genetics, University of Versailles, Garches, France); Ankit Mehta (Baylor University Medical Center at Dallas, Dallas, Texas, USA); Patrick B Deegan (Lysosomal Disorders Unit, Department of Medicine, Cambridge University Hospitals NHS Foundation Trust, Cambridge, UK); Maria Judit Molnar (Institute of Genomic Medicine and Rare Disorders, Semmelweis University Clinical Center, Budapest, Hungary); Damara Ortiz (Department of Pediatrics, UPMC Children’s Hospital of Pittsburgh, Pittsburgh, Pennsylvania, USA); Ana Jovanovic (Mark Holland Metabolic Unit, Northern Care Alliance NHS Foundation Trust, Greater Manchester, UK); Michael Muriello (Department of Pediatrics, Medical College of Wisconsin, Milwaukee, Wisconsin, USA); Bruce A Barshop (Department of Pediatrics, University of California San Diego, La Jolla, California, USA); Virginia Kimonis (Department of Pediatrics, University of California Irvine, Irvine, California, USA); Bojan Vujkovac (Department of Internal Medicine, General Hospital Slovenj Gradec, Slovenj Gradec, Slovenia); Albina Nowak (Department of Endocrinology and Clinical Nutrition, University Hospital Zurich and University of Zurich, Zurich, Switzerland); Tarekegn Geberhiwot (Department of Diabetes, Endocrinology and Metabolism, Queen Elizabeth Hospital Birmingham, Birmingham, UK); Ilkka Kantola (Division of Medicine, Turku University Hospital, Turku, Finland); Jasmine Knoll (Phoenix Children’s Hospital, Phoenix, Arizona, USA); Stephen Waldek (University of Sunderland, Sunderland, UK); Khan Nedd (Infusion Associates, Grand Rapids, Michigan, USA); Amel Karaa (Massachusetts General Hospital for Children, Boston, Massachusetts, USA); David G Warnock (Department of Medicine, Division of Nephrology, The University of Alabama at Birmingham, Birmingham, Alabama, USA).

## Disclosure

ELW had consulting agreements and/or grants with Amicus, Chiesi, 4DMT, Idorsia Pharmaceuticals, Natera, Protalix Biotherapeutics, Sanofi, and Walking Fish Therapeutics. CW has received honoraria for consulting and lecturing from Amicus, Chiesi, Idorsia Pharmaceuticals and Sanofi. Research grants were given from Idorsia Pharmaceuticals and Sanofi to the institution. GP is a full-time employee of Chiesi Farmaceutici. DL is a full-time employee of Chiesi Canada Corp. IK is a full-time employee of Chiesi USA, Inc. RC was a full-time employee of Protalix Biotherapeutics at the time of study conduct and analysis and is now a consultant to Protalix Biotherapeutics. DGW is involved in clinical trials/registries/consulting with Amicus Therapeutics, Chiesi, Idorsia Pharmaceuticals, and Protalix Biotherapeutics.

## Supplementary Material

Supplementary File (PDF) **Figure S1**. Treatment differences in annualized eGFR slope in the overall patient population: primary analysis and post hoc analyses adjusted for proteinuria and calculated using the CKD-EPI creatinine (2009) or the creatinine and cystatin C (2012) equation. **Table S1**. Patient demographics and baseline characteristics.

## Footnotes

Supplementary File (PDF)
**Figure S1**. Treatment differences in annualized eGFR slope in the overall patient population: primary analysis and post hoc analyses adjusted for proteinuria and calculated using the CKD-EPI creatinine (2009) or the creatinine and cystatin C (2012) equation. **Table S1**. Patient demographics and baseline characteristics.
