# Supplementary material for: Impact of Proteinuria on Renal Outcomes in the BALANCE Trial
Source: Kidney Int Rep. 2025 Nov 19;11(2):103691. doi: 10.1016/j.ekir.2025.11.016 (PMC12794064; doi:10.1016/j.ekir.2025.11.016)
Supplement: Supplementary File (PDF) — Figure S1. Treatment differences in annualized eGFR slope in the overall patient population: primary analysis and post hoc analyses adjusted for proteinuria and calculated using the CKD-EPI creatinine (2009) or the creatinine and cystatin C (2012) equation. Table S1. Patient demographics and baseline characteristics. [file mmc1.pdf]

Supplementary material

Table S1. Patient demographics and baseline characteristics

	**Pegunigalsidase alfa (n=51)[a]**	**Agalsidase beta (n=25)**
**Age, years**		
Mean (SD)	44.0 (10.3)	45.2 (9.6)
Min, max	20, 60	18, 58
**Sex, n (%)**		
Male	28 (54.9)	18 (72.0)
Female	23 (45.1)	7 (28.0)
**Race, n (%)**		
White	48 (94.1)	23 (92.0)
**FD classification, n (%)**		
Classic	26 (51.0)	14 (56.0)
Non-classic	25 (49.0)	11 (44.0)
**eGFR (mL/min/1.73 m^2)[b]**		
Mean (SD)	73.9 (20.1)	74.2 (21.0)
Median (min, max)	73.9 (30.2, 125.9)	74.9 (34.1, 107.6)

**eGFR slope (mL/min/1.73 m^2/year)**[b]		
Mean (SD)	−8.0 (6.7)	−8.2 (4.3)
Median (min, max)	−6.4 (−30.5, 6.3)	−7.8 (−20.3, −2.8)
**UPCR (g/g)**		
Mean (SD)	0.44 (0.61)	0.28 (0.49)
Median (min, max)	0.13 (0.02, 3.12)	0.07 (0.02, 2.10)
**UPCR ≤ 0.5 g/g, n (%)**	36 (70.6)	20 (80.0)
Mean (SD)	0.13 (0.12)	0.08 (0.08)
Median (min, max)	0.10 (0.02, 0.45)	0.06 (0.02, 0.32)
**0.5 < UPCR < 1 g/g, n (%)**	8 (15.7)	2 (8.0)
Mean (SD)	0.72 (0.16)	0.64 (0.14)
Median (min, max)	0.71 (0.52, 0.96)	0.64 (0.54, 0.74)
**UPCR ≥ 1 g/g, n (%)**	7 (13.7)	3 (12.0)
Mean (SD)	1.66 (0.76)	1.40 (0.61)
Median (min, max)	1.37 (1.02, 3.12)	1.05 (1.04, 2.10)

[a]In the primary BALANCE analysis, 52 patients were included in the intention-to-treat population for the pegunigalsidase alfa arm; one patient was not included in the post-hoc analyses because they had fewer than four eGFR observations.

[b]eGFR calculated with the Chronic Kidney Disease Epidemiology creatinine (2009) equation.

eGFR, estimated glomerular filtration rate; max, maximum; min, minimum; SD, standard deviation; UPCR, urine protein-to-creatinine ratio.

Figure S1. Treatment differences in annualized eGFR slope in the overall patient population: primary analysis and *post hoc* analyses adjusted for proteinuria and calculated using the CKD-EPI creatinine (2009) or the creatinine and cystatin C (2012) equation

Analysis/2-stage model	eGFR slope (mL/min/1.73 m^2/year) Pegunigalsidase alfa n = 51	eGFR slope (mL/min/1.73 m^2/year) Agalsidase beta n = 25	Treatment difference (95% CI)
**Primary analysis: quantile regression**[a]			
Median (CKD-EPI creatinine [2009] equation)	−2.5	−2.2	−0.4 (−2.4, 1.7)
***Post hoc* analysis: quantile regression**[b]			
Median (CKD-EPI creatinine [2009] equation)	−1.2	−1.3	0.1 (−2.0, 2.2)
Median (CKD-EPI creatinine and cystatin [2012] equation)	−0.6	−1.3	0.8 (−1.3, 2.8)
***Post hoc* analysis: ANCOVA**[b]			
Mean (CKD-EPI creatinine [2009] equation)	−2.0	−3.1	1.1 (−2.0, 4.2)
Mean (CKD-EPI creatinine and cystatin [2012] equation)	−2.3	−2.7	0.4 (−2.6, 3.4)

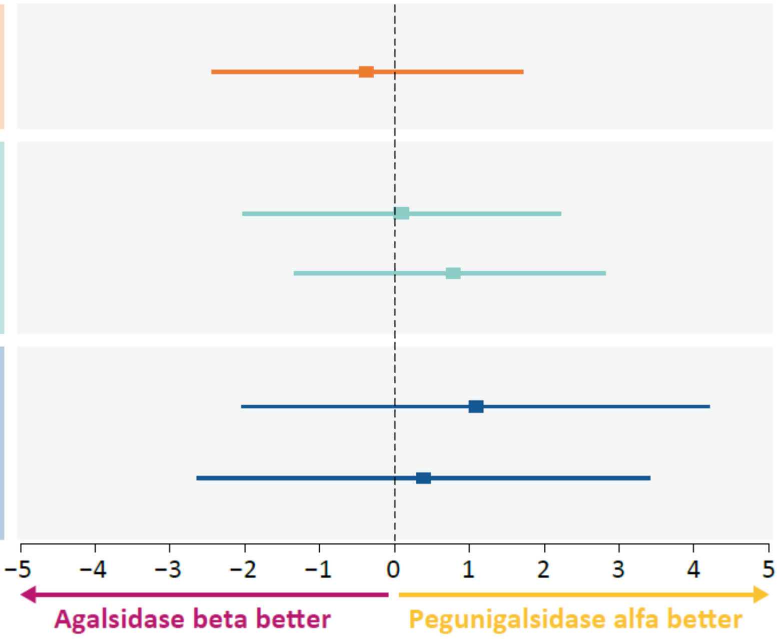

[a]eGFR slope not adjusted for proteinuria.

[b]eGFR slope of each patient as a dependent variable, and treatment arm and UPCR as covariates.

CI, confidence interval; CKD-EPI, Chronic Kidney Disease Epidemiology; eGFR, estimated glomerular filtration rate; UPCR, urine protein-to-creatinine ratio
